# Supplementary material for: Local correlations necessitate waterfalls as a connection between quasiparticle band and developing Hubbard bands
Source: Nat Commun. 2025 Jan 2;16:255. doi: 10.1038/s41467-024-55465-7 (PMC11695606; doi:10.1038/s41467-024-55465-7)
Supplement: Supplementary file 1 — Supplementary Information [file 41467_2024_55465_MOESM1_ESM.pdf]

Supplementary Information to "Local correlations necessitate waterfalls as a connection between quasiparticle band and developing Hubbard bands"

Juraj Krsnik[1,2,*] and Karsten Held[1,†]

[1] *Institute of Solid State Physics, TU Wien, 1040 Vienna, Austria*
[2] *Department for Research of Materials under Extreme Conditions, Institute of Physics, 10000 Zagreb, Croatia*

(Dated: November 28, 2024)

In this Supplementary Information, we discuss the impact of non-local correlations such as spin fluctuations, which lead to the momentum dependence of the self-energy, on the waterfall structure in the spectral function. In particular, we employ an extension of dynamical mean-field theory (DMFT), namely the dynamical vertex approximation (DΓA), and we compare these results with our DMFT findings. We find that while the non-local correlations may further corroborate the waterfall-like features, their origin is still that of local correlations already accounted for by DMFT. Namely, the waterfall appears whenever $\partial \mathrm{Re}\Sigma(\mathbf{k},\omega)/\partial\omega = 1$ and at the momentum where $\varepsilon_{\mathbf{k}} + \mathrm{Re}\Sigma(\mathbf{k},\omega)$ lies on the ω line.

SUPPLEMENTARY NOTE 1: NON-LOCAL CORRELATION EFFECTS ON WATERFALLS - A COMPARATIVE STUDY OF DMFT AND DΓA

To study the effects of non-local correlations on the waterfall-like features in the spectral function, we extend our dynamical mean-field theory (DMFT) results by utilizing the dynamical vertex approximation (DΓA) [1, 2]. In

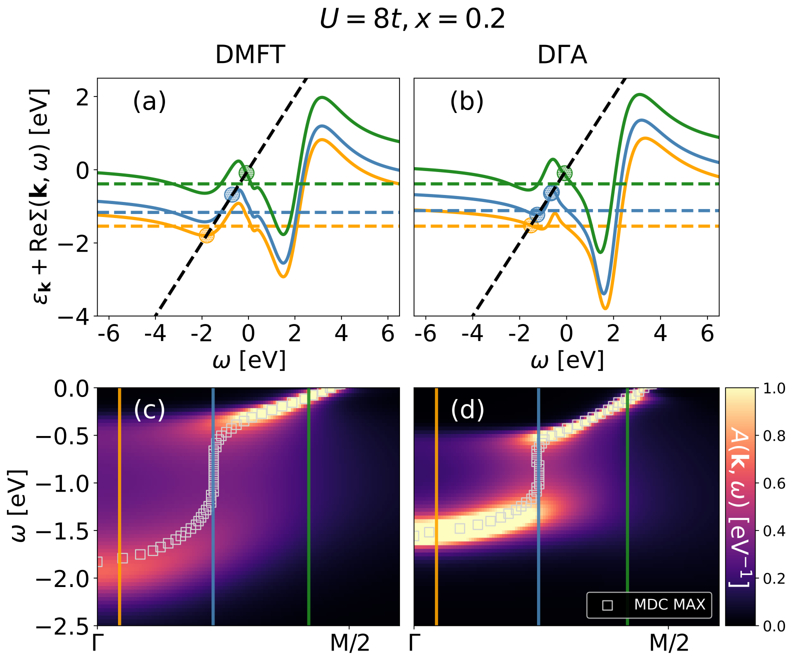

Supplementary Figure 1. **Graphical solutions of the pole equation and spectral functions for 20% hole doping in DMFT and DΓA.** (a, b) Graphical solution for the poles of the Green's function in Eq. (2) of the main text as the crossing point (colored circles) between $\varepsilon_{\mathbf{k}} + \mathrm{Re}\Sigma(\mathbf{k},\omega)$ (solid lines in three colors for the three $\mathbf{k}$ points indicated by vertical lines in the bottom panel) and ω (black dashed line) in DMFT (left column) and DΓA (right column); the colored dashed lines denote $\varepsilon_{\mathbf{k}}$ for the same three momenta. (c, d) $\mathbf{k}$-resolved spectral functions $A(\mathbf{k},\omega)$ along the nodal direction $\Gamma = (0,0)$ to $\mathrm{M} = (\pi,\pi)$ for $U = 8t$ and 20% hole doping. Also plotted are the maxima of corresponding momentum distribution curves (MDC MAX, grey squares).

* juraj.krsnik@tuwien.ac.at
† held@ifp.tuwien.ac.at

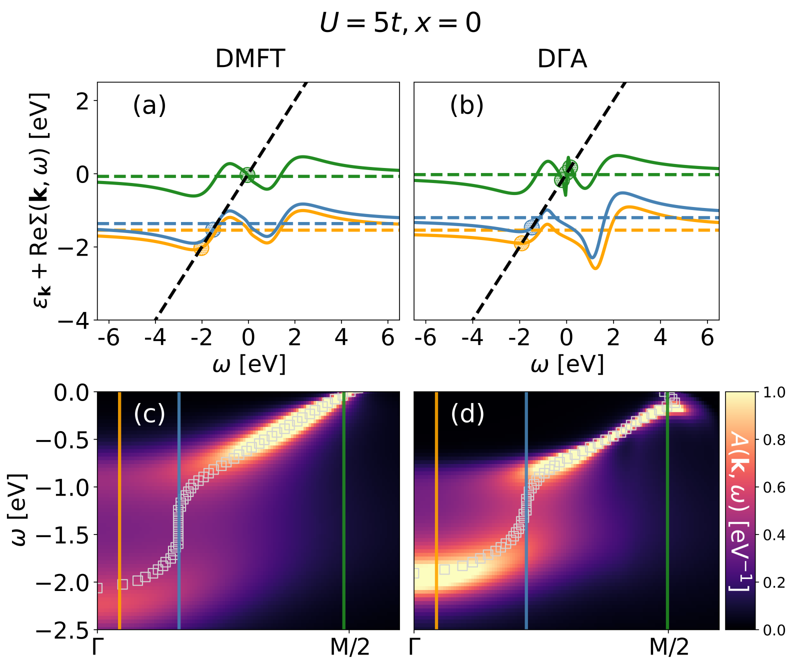

Supplementary Figure 2. **Graphical solutions of the pole equation and spectral functions at half-filling in DMFT and DΓA.** (a, b) Graphical solution for the poles of the Green's function in Eq. (2) of the main text as the crossing point (colored circles) between $\varepsilon_{\mathbf{k}} + \mathrm{Re}\Sigma(\mathbf{k}, \omega)$ (solid lines in three colors for the three $\mathbf{k}$ points indicated by vertical lines in the bottom panel) and ω (black dashed line) in DMFT (left column) and DΓA (right column); the colored dashed lines denote $\varepsilon_{\mathbf{k}}$ for the same three momenta. (c, d) $\mathbf{k}$-resolved spectral functions $A(\mathbf{k}, \omega)$ along the nodal direction $\Gamma = (0, 0)$ to $\mathrm{M} = (\pi, \pi)$ for $U = 5t$ and half-filling. Also plotted are the maxima of corresponding momentum distribution curves (MDC MAX, grey squares).

particular, we use the ladder version of DΓA as implemented in the `DGApy` code [3], which incorporates the non-local effects of spin fluctuations, yielding a momentum-dependent self-energy $\Sigma(\mathbf{k}, \omega)$. As in the main text, we study the Hubbard model with nearest neighbor hopping t, Coulomb interaction U, and doping x, corresponding to filling of $n = 1 - x$ electrons per site.

In Supplementary Fig. 1, we present a comparison between the DMFT and DΓA results for the case with $U = 8t$ and 20% hole doping for which the (almost vertical) waterfall-like structure is clearly present in the DMFT spectrum, see Supplementary Fig. 1(c) and Figs. 2(c, g) in the main text. The nearest neighbor hopping is $t = 0.3894$ eV and the temperature is room temperature $T = t/15$. The DΓA spectrum in Supplementary Fig. 1(d) features the vertical waterfall-like structure even more prominently, with the sharp drop of the momentum distribution curves (MDCs) maxima at roughly the same wave vector (denoted in blue) as in DMFT. Compared to the DMFT case, we now have some additional renormalization of both the quasiparticle (QP) band and the Hubbard band, so the waterfall starts and ends at slightly different energies than in DMFT. This further supports the notion that the waterfall is indeed a feature connecting the QP and Hubbard bands. That is, while the DΓA spectrum in Supplementary Fig. 1(d) suggests that the spin fluctuations may further enhance the clarity of waterfall-like effects in the spectra, these features still originate from the local correlations already captured by DMFT.

To further elaborate on this point, in Supplementary Figs. 1(a) and (b) we compare the graphical solutions of the pole equation for three different momenta (discriminated by color) within DMFT and DΓA, respectively. As explained in the main text, the waterfall in the DMFT spectrum appears at the wave vector for which $\varepsilon_{\mathbf{k}} + \mathrm{Re}\Sigma(\omega)$ lies on the ω line, see Supplementary Fig. 1(a). In the DΓA case, shown in Supplementary Figs. 1(b) and (d), the same reasoning applies, except that now the self-energy gains a momentum dependence. In particular, we again note that for the wave vector (denoted in blue) where the waterfall appears in the spectrum in Supplementary Fig. 1(d) $\varepsilon_{\mathbf{k}} + \mathrm{Re}\Sigma(\mathbf{k}, \omega)$ lies on the ω line in Supplementary Fig. 1(b). This then points to the same mechanism behind the waterfall formation as in DMFT and also explains why the waterfall appears to be shorter: $\partial \mathrm{Re}\Sigma(\mathbf{k}, \omega)/\partial\omega = 1$ holds in a smaller energy range.

To even better emphasize the differences between DMFT and DΓA, in Supplementary Fig. 2 we compare the DMFT and DΓA spectra for $U = 5t$ at half-filling. For these parameters, we also anticipate a vertical waterfall-like structure within DMFT, see Fig. 1(j) in the main text and Supplementary Fig. 2(c). Without doping, strong spin fluctuations

open a gap at low energies in the DΓA spectrum as seen in Supplementary Fig. 2(d). Correspondingly, we see that for these momenta $\mathrm{Re}\Sigma(\mathbf{k},\omega) \sim -\omega$ no longer holds at small frequencies. Nevertheless, even though we have a large effect of non-local correlations at low energies, we still preserve the DMFT waterfall-like structure appearing at much larger energies. Again, the waterfall is at the similar wave vector as in DMFT, and $\varepsilon_{\mathbf{k}} + \mathrm{Re}\Sigma(\mathbf{k},\omega)$ on the ω line, see Supplementary Fig. 2(b).

[1] A. Toschi, A. A. Katanin, and K. Held, Dynamical vertex approximation; A step beyond dynamical mean-field theory, Phys Rev. B **75**, 45118 (2007).
[2] G. Rohringer et al., Diagrammatic routes to nonlocal correlations beyond dynamical mean field theory, Rev. Mod. Phys. **90**, 25003 (2018).
[3] P. Worm, DGApy, https://github.com/PaulWorm/DGApy (2023).
