# Supplementary material for: Local correlations necessitate waterfalls as a connection between quasiparticle band and developing Hubbard bands
Source: Nat Commun. 2025 Jan 2;16:255. doi: 10.1038/s41467-024-55465-7 (PMC11695606; doi:10.1038/s41467-024-55465-7)
Supplement: Supplementary file 2 — Description of Additional Supplementary Files [file 41467_2024_55465_MOESM2_ESM.pdf]

Description of Additional Supplementary Files

File name: Supplementary Movie 1

Description: Splitting-off of the lower Hubbard band from the central quasiparticle band with increasing interaction U and their waterfall-like connection. The false color denotes the DMFT spectrum $A(\mathbf{k},\omega)$. The waterfalls are most clearly identifiable as a sharp drop in the momentum distribution curve maxima (MDC MAX), similar to experiment. Also shown are the energy distribution curve maxima (EDC MAX).
