# Supplementary material for: Local correlations necessitate waterfalls as a connection between quasiparticle band and developing Hubbard bands
Source: Nat Commun. 2025 Jan 2;16:255. doi: 10.1038/s41467-024-55465-7 (PMC11695606; doi:10.1038/s41467-024-55465-7)
Supplement: Supplementary file 4 — Author Checklist [file 41467_2024_55465_MOESM4_ESM.pdf]

nature portfolio

Author Checklist

NCOMMS-24-45892A

Please check the items below carefully and add a response in each row of the table to indicate the changes that you have made. Please also check through any additional marked-up edits we may have provided within the manuscript file.

Abstract and editor's summary

Our guidance:	Your response:
Your paper will be accompanied by the following editor's summary. Please let us know if there are any inaccuracies: 'In angle-resolved photoemission spectra, nearly vertical energy-momentum dispersions, often termed waterfalls, can occur at high energies in materials including nickelates and cuprates. Using dynamical mean-field theory, the authors show that these features naturally emerge when a Hubbard band develops and splits off from the central quasiparticle band.'	The summary is fine.
The abstract — which should be roughly 150 words long and contain no references — should serve both as a general introduction to the topic and a non-technical summary of your main results and their implications. It should contain a brief account of the background and rationale of the work, followed by a statement of the main conclusions introduced by the phrase 'Here we show' or some equivalent phrase. Because we hope that researchers in a wide range of disciplines will be interested in your work, the abstract should be as accessible as possible, explaining essential but specialized terms concisely. We encourage you to show your abstract to colleagues outside of your direct field of expertise to uncover any problematic concepts.	The abstract is appropriately written with a “Here we show” statement in the last but one sentence. We extended the abstract somewhat to better convey our summary.
Please edit the title so that it is 15 words or fewer and does not include punctuation.	Please see the next point.
According to our journal´s guidelines, the title must be descriptive, informative, appropriate, and readily intelligible to any scientist. We feel that the current title does not satisfy these requirements. We therefore kindly ask that you remove the metaphor from the title and create a more descriptive title that references the underlying physics.	We changed the title to “Local correlations necessitate waterfalls as a connection between quasiparticle and developing Hubbard bands”.
Please include a concluding sentence in the abstract.	The last two sentences are concluding.

Author information

Our guidance:	Your response:
We ask that you consult with your coauthors to ensure that all names, affiliations, and titles are represented correctly. Note that if any authors are added or removed after this point then all authors will be requested to provide approval documentation that could potentially delay the production of your paper.	All names, affiliations, and titles are represented correctly. We added one affiliation.

Ensure affiliations are appropriately labeled and featured sequentially and in ascending order (1,2,3,... or a,b,c...). Please ensure all corresponding authors are marked with a specific symbol and include their emails.<br>Similarly, if you have “equally contributing” or “joint supervision” authors, use a specific symbol to mark them and not a number.	Affiliations are appropriately labeled and featured sequentially and in ascending order. All corresponding authors are marked with a specific symbol and their emails are included.
Please ensure the author contributions section mentions each author's initials at least once with their contributions to the work. Authors with the same initials must be differentiated in the statement.	Each author’s initials are mentioned at least once. There are no authors with the same initials.

Article structure

Our guidance:	Your response:
We can accommodate up to 10 display items (Figures or Tables) in the main article. Each Figure and Table must fit easily within an A4 page (210 x 297 mm). Please ensure that the number and size of your Figures and Tables fulfil these requirements to avoid any delay in the acceptance of your article.	We have 4 Figures and each fit within an A4 page.
Ensure main Figures are uploaded as separate individual files. Each figure file must contain all intended panels labelled and displayed as intended and fit entirely on a single page. Do NOT include legends within the figure files, as these must be in the main manuscript.<br><br>Supplementary Figures must be all contained in the Supplementary Information PDF and do NOT need to be uploaded separately.	All Figures are uploaded as separate individual files and satisfy the required conditions.
**Please ensure your main manuscript file includes the following sections, in this order:**<br>*Title*<br>*Author list*<br>*Affiliations*<br>*Abstract*<br>*Introduction*<br>*Results*<br>*Discussion (optional)*<br>*Results and Discussion (optional)*<br>*Methods*<br>*Data Availability*<br>*Code Availability (if relevant)*<br>*References*<br>*Acknowledgements*<br>*Author Contributions Statement*<br>*Competing Interests Statement*<br>*Tables*<br>*Figure Legends/Captions (for main text figures)*<br><br>We do not edit Supplementary Information files; they will be uploaded with the published article as they are submitted with the final version of your manuscript. Any tracked changes should be removed from the file and the file should be provided as a PDF file. Supplementary Figures do not need to be provided separately.	We added Introduction, Results, and Discussion sections. For better readability we have additional subsections.<br><br>We placed the Data Availability, Code Availability, References, Acknowledgements, Author Contributions, and Competing Interests sections in the requested order.
Please consider making the raw data underlying your figures available to readers as source data files.	Please see the next point.
Within the Source Data file, the relevant raw data from each figure or table (in the main manuscript and in the Supplementary Information) should be represented by a single sheet in an Excel document, or a single .txt file or other file type in a zipped folder. An example of the Source Data file is available demonstrating the correct format:<br><br>https://www.nature.com/documents/ncomms-example-source-data.xlsx<br><br>The file should be labelled 'Source Data', with the title and a brief description included in your response here, and should be mentioned in	All relevant raw data used in the manuscript can be find in a repository hosted by TU Wien, which we now cite in the Data availability section.

all relevant figure legends using the template text below:<br><br>'Source data are provided as a Source Data file.'<br><br>A reference to the source data file should be added in the 'Data Availability' section, using the text "Source data are provided with this paper".	

Main text

Our guidance:	Your response:
Please do not use italics, bold font, underlining or speech marks/quotation marks except in headings unless required for technical terms (in both the main text and the display items).	We removed italics, bold font, and speech marks/quotation marks throughout the main text, though we keep them for the metaphor where we find it appropriate.
Please make sure that mathematical terms throughout your manuscript and Supplementary Information (including in figures, figure axes, and legends) conform strictly to the following guidelines. Equations must be supplied in editable format, and not as images. Scalar variables (e.g. x, V, χ) must be typeset in italic, whereas multi-letter variables and functions (e.g. log) must be formatted in roman. Vectors (such as the wavevector k or the magnetic field vector B) must be typeset in bold without italics.	All mathematical terms are properly written.
Please remove terms like "excellent" and "perfectly" from the main text.	We replaced "excellent" with (very) good, and "perfectly" with well in the main text.
Please ensure that atomic orbital notations (sp, d, etc.) are typeset in italics, whereas all accompanying superscripts/subscripts are typeset in Roman.	All atomic orbital labels are typeset as requested.
Supplementary Information:	
Please change the title to Supplementary Information.	We changed the title to Supplementary Information as requested.
In the Supplementary Information file and the main manuscript text, supplementary items must be labelled and cited using only the following formats: Supplementary Figure 1, Supplementary Table 1, Supplementary Methods, Supplementary Note 1, Supplementary Discussion, and Supplementary References. Please note the use of "Supplementary" and that we do not use the "S" prefix. In particular, please make the sections of the SI into Supplementary Notes, e.g. "Supplementary Note 1: …".	Since we have only one section in Supplementary Information, we dropped the notation "Supplementary Note" completely.
Please label and refer to figures using the form "Supplementary Fig. X" instead of "Fig. SX".	We now use "Supplementary Fig. X" when referring to figures.
Please amend currently used citations to Supplementary Information in the main text, e.g. "See Supplementary Note 1", "See Supplementary Fig.1", etc.	We now use Supplementary Fig. 1 and 2 when referring to Supplementary Figures in the main text.
Please label equations in the Supplementary Information file as (1), (2), etc. and refer to them in the text as Supplementary Eq. (1), Supplementary Eq. (2), etc.	There are no equations in the Supplementary Information file.

Figures and Tables

Our guidance:	Your response:
Please see the guidelines linked below for detailed instructions about how your figures should be prepared. Following these instructions will reduce the chances of delays should we need to request replacement artwork from you at a later stage.<br>https://www.nature.com/documents/NRJs-guide-to-preparing-final-artwork.pdf	We checked the instructions and made sure that our figures comply with them.

Shadings or symbols in graphs must be defined in some fashion. We prefer that you use a key within the image; do not include colored symbols in the legend/caption.	We defined all the necessary shadings and symbols. In particular, we added a description of the shaded areas in the top row of Fig. 1 and to the circles and the colored dashed lines in the second row of Fig.1.
All figure legends must include a brief title that summarises the whole figure.	For each figure we included a brief title at the beginning of the figure legend denoted in bold.
Any abbreviations, symbols or colours present in your figures must be defined in the associated legends.	We have done so.
Please define all plotted data sets, including each color, solid line, and dashed line. In particular, please define the dashed colored lines in the Figures and Supplementary Figures.	We have done so.
Please ensure that the panel labels in your Figures and Figure captions are consistent.	They are consistent.
In the Figure 3 caption, please define what is meant by "a sufficiently low temperature".	It is defined as 100/t.
Please be sure to include axis labels and units for all graphs, including spectral function plots.	We have done so.

Data and Code

Our guidance:	Your response:
Nature journals strongly support public availability of data and code. Please deposit the data and code used in your paper into a public data repository, or alternatively, present the data as Supplementary Information. If data can only be shared on request, please explain why in your Data Availability Statement, and also in the correspondence with your editor.<br><br>Please note that for some data types, deposition in a public repository is mandatory. Any restrictions on sharing of these data types must be clearly indicated in the statement and discussed with the editor. More information on our data deposition policies and available repositories can be found here:<br><br>https://www.nature.com/nature-research/editorial-policies/reporting-standards#availability-of-data	All the data and code used in the paper are publicly available and can be found as described in the main text.
All published manuscripts reporting original research in Nature Portfolio journals must include a data availability statement, within the Methods and under the heading 'Data Availability'.<br><br>The data availability statement must make the conditions of access to the “minimum dataset” that are necessary to interpret, verify and extend the research in the article, transparent to readers. We ask that you don’t use phrases like ‘available on reasonable request’ but instead specify any restrictions to accessing your data as described below.<br><br>This minimum dataset may be provided through deposition in public community/discipline-specific repositories, custom proprietary repositories or general repositories like Figshare, Zenodo and Dryad. Providing large datasets in supplementary information is strongly discouraged and the preferred approach is to make data available in repositories. Please see https://www.springernature.com/gp/authors/research-data-policy/recommended-repositories for a list of recommended repositories.<br><br>If DOIs are provided, we also strongly encourage including these in the Reference list (authors, title, publisher (repository name), identifier, year).<br><br>The Data Availability Statement should also reference any source data published alongside the paper.<br><br>For clinical datasets or third party data, please ensure that the Data Availability statement adheres to our policy	Please see the last point under the Article Structure.

(https://www.nature.com/nature-research/editorial-policies/reporting-standards#availability-of-data)<br><br>If data are unavailable, please indicate the exact reasons why data cannot be made available in a suitable public repository or upon request, including any conditions related to ethical approval, consent from study subjects, commercial or legal restrictions, etc.<br><br>For data that are available under restricted access, the Data Availability statement must specify<br>- the reasons for access restrictions<br>- what the restrictions are<br>- how one can get access to the data<br>- who to contact to request access<br>- any restrictions on who the data can be made available to or for which purpose<br>- the expected timeframe for response to access requests<br>- for how long the data will be available once access has been granted.	
Please use the following template to provide all the information stated above:<br><br>The XX data generated in this study have been deposited in the YY database under accession code ZZ [add hyperlink here]. The XX data are available under restricted access for {insert reason}, access can be obtained by {explain how}. The raw XX data are protected and are not available due to data privacy laws. The processed XX data are available at YY. The XX data generated in this study are provided in the Supplementary Information/Source Data file. The XX data used in this study are available in the YY database under accession code ZZ [Add hyperlink here].	All data are in the same repository, and the repository is properly referenced.
We notice that you have deposited your code in a Github repository, which we fully support. We strongly encourage you in addition to make your code citable by obtaining a DOI for the Github repository in order to provide a permanent reference to the version of the code used in this study and improve reproducibility. This can be done by linking the repository to Zenodo, following the instructions here: https://guides.github.com/activities/citable-code/ Please cite the Github repository in your manuscript text or Code Availability statement and in your reference list: authors, title (this paper), repository name, DOI identifier, year. Alternatively, you can deposit the code in Gigantum or Code Ocean for the same purpose.	All used Github repositories are cited.
Please make sure all links are functional as we need to check them and ensure the data has been deposited correctly.	All links are functionals and the data has been deposited correctly.
Please explain which data is contained under which link and, if possible, reduce the number of links.	Everything is properly explained and the number of links reduced to a minimum level.

References

Our guidance:	Your response:
All references must be cited in numerical order. The reference list will be formatted according to the Nature style by our journal production team, however please ensure that references contain all of the information required, eg:<br><br>Kurumada, S., Takamori, S. & Yamashita, M. An alkyl-substituted aluminium anion with strong basicity and nucleophilicity. <i>Nat. Chem.</i> **12**, 36–39 (2020).	All references are cited in numerical order and contain all of the information required.
Supplementary References should appear at the end of the Supplementary Information file, and must be self-contained and numbered from 1. References mentioned in both the main text and the Supplementary Information should be part of both reference lists so that the Supplementary Information does not refer to the reference list in the main paper and vice versa.	All Supplementary References appear at the end of the Supplementary Information file, are self-contained and numbered from 1.

Please check if any preprints are now published papers and update those citations.	All the papers that got published are now cited instead of preprints.

End matter

Our guidance:	Your response:
Nature Portfolio defines Competing Interest (CI) as financial and non-financial interests (including but not limited to funding, employment, stocks, shares, patents, personal or professional relationships with individuals or institutions, and unpaid membership advocacy) that could be perceived to directly undermine the objectivity, integrity, and value of a publication, or could be seen as having an influence on the judgments and actions of authors with regard to objective data presentation, analysis, and interpretation.<br><br>Please thoroughly review our policy on Competing Interests and include a detailed statement both in your final manuscript file and in our manuscript tracking system. Please ensure the statements are identical in both. Be specific about how each point stated relates to the research and list applicable author initials, and/or patent numbers.<br><br>If there are no competing interests, a negative statement must be included.<br>https://www.nature.com/nature-research/editorial-policies/competing-interests	There are no competing interests.
Please confirm that all relevant funding awarded to each author is described in the Acknowledgements section. List each grant number, followed by the initials of the author who received it.	We have identified each grant with initials.

Preparing your manuscript files

Our guidance:	Your response:
Unless otherwise stated please limit individual file sizes to approximately 30MB. We strongly encourage the use of repositories for large datasets or source data due to size considerations.	All files are smaller than 30 MB.
Please supply the main manuscript file in either Microsoft Word or LaTeX format	Done.
Please provide figures as individual vector files with editable text. Acceptable file types for figures are .ai, .eps, .pdf, .ppt or Chem Draw for fully editable vector-based art. For detailed guidance on figure preparation, see https://www.nature.com/documents/aj-artworkguidelines.pdf	All figures are pdf.
Please supply legends for each Supplementary Movie/Audio/Data file in your response here (not in the Supplementary Information file). Please label each files as Supplementary Movie/Audio/Data 1, etc.	There is only one movie. The legend is:<br><br>Splitting-off of the lower Hubbard band from the central quasiparticle band with increasing interaction U and their waterfall-like connection. The false color denotes the DMFT spectrum $A(\mathbf{k},\omega)$. The waterfalls are most clearly identifiable as a sharp drop in the momentum distribution curve maxima (MDC MAX), similar to experiment. Also shown are the energy distribution curve maxima (EDC MAX).
The use or adaptation of previously published images is strongly discouraged. If this is unavoidable, please request the necessary rights documentation to re-use such material from the relevant copyright holders and return this to us when you submit your revised manuscript. Please check whether your manuscript or Supplementary Information contain third-party images, such as figures from the literature, stock photos, clip art or commercial satellite and map data.	We did not use previously published figures, just plotted digitalized data points from previous figures.

If any elements of your submitted work have been created with BioRender you will need to ensure you have obtained a publication license from BioRender, adhering to the user requirements as outlined within the license. The reference for BioRender created graphics should be present in the accompanying legend of the display material it is present in.<br><br>A copy of the publication license should be uploaded to our system as a related manuscript file upon resubmission.<br><br>For more information please see the BioRender knowledge article here: https://help.biorender.com/hc/en-gb/articles/21283116932765-CC-BY-publishing-and-reader-permissions<br><br>For more information on what constitutes ownership by a third party, please contact our Editorial Assistant at naturecommunications@nature.com	

Forms to complete

Our guidance:	Your response:
**Editorial Policy Checklist**<br>Please update and upload a final version of the Editorial Policy Checklist with your revised manuscript files. A blank Editorial Policy Checklist can be found via the link below. Note that this form is a dynamic 'smart pdf' and must be downloaded and completed in Adobe Reader.<br><br>Please update your current checklist or download from:<br><br>https://www.nature.com/documents/nr-editorial-policy-checklist.zip	We have done so.

You will need to upload:

Editorial Policy Checklist	included
Completed Third Party Rights Table (if relevant)	not relevant
A completed copy of this checklist	included
The main manuscript file in either Microsoft Word or LaTeX format	included
Separate Figure files	included
Inventory of Supporting Information	included
A Supplementary Information file	included
Supplementary Table, Video, Audio, Data, or Software files	one movie included
